# Cytotoxicity and Glycan-Binding Properties of an 18 kDa Lectin Isolated from the Marine Sponge *Halichondria okadai*

**DOI:** 10.3390/toxins4050323

**Published:** 2012-04-30

**Authors:** Ryo Matsumoto, Yuki Fujii, Sarkar M. A. Kawsar, Robert A. Kanaly, Hidetaro Yasumitsu, Yasuhiro Koide, Imtiaj Hasan, Chihiro Iwahara, Yukiko Ogawa, Chang Hun Im, Shigeki Sugawara, Masahiro Hosono, Kazuo Nitta, Jiharu Hamako, Taei Matsui, Yasuhiro Ozeki

**Affiliations:** 1 Laboratory of Glycobiology and Marine Biochemistry, Department of Genome System Science, Graduate School of NanoBio Sciences, Yokohama City University, 22-2 Seto, Kanazawa-ku, Yokohama 236-0027, Japan; Email: akagiwashizuten@yahoo.co.jp (R.M.); yfujii@niu.ac.jp (Y.F.); akawsarabe@yahoo.com (S.M.A.K.); kanaly@yokohama-cu.ac.jp (R.A.K.); hyasumit@yokohama-cu.ac.jp (H.Y.); y.koide-haliotis@kzd.biglobe.ne.jp (Y.K.); hasanimtiaj@yahoo.co.uk (I.H.); c_hiro_iwahara@yahoo.co.jp (C.I.); 2 Divisions of Functional Morphology and Microbiology, Department of Pharmacy, Faculty of Pharmaceutical Science, Nagasaki International University, 2825-7 Huis Ten Bosch, Sasebo, Nagasaki 859-3298, Japan; Email: yogawa@niu.ac.jp; 3 Laboratory of Carbohydrate and Protein Chemistry, Department of Chemistry, Faculty of Science, University of Chittagong, Chittagong-4221, Bangladesh; 4 Division of Cell Recognition Study, Institute of Molecular Biomembrane and Glycobiology, Tohoku Pharmaceutical University, 4-4-1 Komatsusima, Aoba-ku, Sendai 981-8558, Japan; Email: ich@kdis.ne.jp (C.H.I.); ssuga@tohoku-pharm.ac.jp (S.S.); mhosono@tohoku-pharm.ac.jp (M.H.); knitta@tohoku-pharm.ac.jp (K.N.); 5 Department of Biology, Fujita Health University, Toyoake, Aichi 470-1192, Japan; Email: jihamako@fujita-hu.ac.jp (J.H.); tmatsui@fujita-hu.ac.jp (T.M.)

**Keywords:** cytotoxicity, frontal affinity chromatography technology, glycoprotein, Japanese black sponge (*Halichondria okadai*), lectin

## Abstract

A divalent cation-independent lectin—HOL-18, with cytotoxic activity against leukemia cells, was purified from a demosponge, *Halichondria okadai*. HOL-18 is a 72 kDa tetrameric lectin that consists of four non-covalently bonded 18 kDa subunits. Hemagglutination activity of the lectin was strongly inhibited by chitotriose (GlcNAcβ1-4GlcNAcβ1-4GlcNAc), fetuin and mucins from porcine stomach and bovine submaxillary gland. Lectin activity was stable at pH 4–12 and temperatures lower than 60 °C. Frontal affinity chromatography with 16 types of pyridylaminated oligosaccharides indicated that the lectin had an affinity for *N*-linked complex-type and sphingolipid-type oligosaccharides with *N*-acetylated hexosamines and neuramic acid at the non-reducing termini. The lectin killed Jurkat leukemia T cells and K562 erythroleukemia cells in a dose- and carbohydrate-dependent manner.

## 1. Introduction

Lectins are carbohydrate-binding proteins that are present among various living organisms. They have many functions including cell growth regulation, anti-infectious properties and support of natural immunity through their binding to specific oligosaccharides to form glycoconjugates (glycoproteins and glycosphingolipids) [1]. At the same time, administration of some lectins isolated from plants and lower animals have been shown to kill culture cells derived from higher animals [2,3]. Sponges, which are known to be one of the oldest living groups of organisms, produce different types of biological molecules that regulate their growth through symbiotic relationships with microorganisms. In regard to this, unique chemicals that have attractive activities such as okadaic acid and halichondrin B, which are known to act as a phosphatase inhibitor and as an anticancer agent, respectively, are produced by symbiotic microorganisms that are isolated from the Japanese black sponge *Halichondria okadai* [4,5]. 

Many types of lectins are produced in different species of sponges whose habitats range from tidal zones to the deep sea. *N*-acetyl D-galactosamine (D-GalNAc)-binding lectins, known as galectins, are a representative class of animal lectins that have been isolated from the demosponges *Geodia cydonium* [6] and *Suberites domuncula* [7]. Additionally, a 14 kDa *N*-acetyl amino-carbohydrate-specific lectin has been obtained from *Axinella corrugate* [8]. In another study, cDNAs encoding a polypeptide similar to the lipopolysaccharide-binding lectin of the horseshoe crab were isolated from *S. domuncula* and *Ephydatia fluviatilis* and interestingly were expressed during sponge development [9,10]. In the Japanese black sponge, *H. okadai* mentioned above, three lectins (HOL-I, HOL-II and HOL-30) were purified using affinity chromatography on bovine submaxillary mucin-Sepharose, acid-treated Sepharose 4B and lactosyl-agarose, respectively [11,12]. HOL-I, a 21-kDa polypeptide, recognized *N*-acetyl amino-carbohydrates such as *N*-acetyl D-glucosamine (D-GlcNAc) and D-GalNAc while HOL-II, a 42-kDa polypeptide, recognized D-Gal and type-1 *N*-acetyllactosamine (Galβ1-3GlcNAc). On the other hand, HOL-30, a 30 kDa β-galactoside-binding lectin with a galectin-like primary structure, was indicated to have an affinity to type-1 and type-2 (Galβ1-4GlcNAc) *N*-acetyllactosamines of branched complex-type oligosaccharides.

Recently, a preliminary investigation suggested the presence of another lectin in *H. okadai*, which has a smaller molecular mass than HOL-I, HOL-30, and HOL-II and which demonstrates a binding affinity to chitotriose (GlcNAcβ1-4GlcNAcβ1-4GlcNAc). If the isolation of the lectin is successful and the details of its biochemical properties using a glycome approach are accomplished, knowledge of a new sponge lectin may be added to the growing literature in this field. Frontal affinity chromatography technology (FACT) is a method by which the glycan-binding specificity of lectins may be evaluated by using various types of pyridylamino group (PA)-labeled oligosaccharides on an affinity column whereby the delay in the elution of the lectin is indicative of its binding affinity [13,14]. This information in regard to the binding property of the lectin will aid in the understanding of the novel characters of each lectin. In this study, the specific glycan-binding properties and glycan-dependent cytotoxicity of an 18 kDa lectin isolated from a Japanese black sponge were evaluated.

## 2. Materials and Methods

### 2.1. Animal and Chemicals

The demosponge *H. okadai* was collected from the tidal zone of the Zushi coast, Kanagawa Prefecture, Japan. The samples were used immediately or stored at −80 °C until studied.

Lactose, melibiose, D-galactose (D-Gal), D-glucose (D-Glc), D-mannose (D-Man) and L-fucose (L-Fuc) were purchased from Wako Pure Chemical Co. Inc., Japan. Chitobiose and chitotriose were purchased from Carbosynth Ltd., UK. Methyl α-*N*-actyl D-glucosaminide (Me-α-GlcNAc), methyl β-*N*-actyl D-glucosaminide (Me-β-GlcNAc), methyl α-*N*-actyl D-galactosaminide (Me-α-GalNAc) and methyl β-*N*-actyl D-galactosaminide (Me-β-GalNAc) were purchased from Toronto Research Chemicals Inc., Canada. Glycoproteins, bovine submaxillary mucin (BSM), fetuin, porcine stomach mucin (PSM) and standard protein markers for gel permeation chromatography were purchased from Sigma Chemical Co., USA. Chitotriose-agarose, lactosyl-agarose, horseradish peroxidase-conjugated lectins; wheat germ agglutinin (HRP-WGA), Concanavalin A (HRP-Con A) and *Ricinus communis* agglutinin (HRP-RCA 120) and a protease inhibitor mixture were purchased from Cosmo Bio Co. Ltd., Japan. Superdex 75, Sephadex G-75, the CM5 sensor chip and a ligand coupling kit were obtained from GE Healthcare, USA. The bicinchoninic acid (BCA) kit was obtained from Pierce Co. Ltd., USA. Pre-stained standard protein marker mixture (EzStandard PrestainBlue), |3,3′,5,5′-tetramethylbenzidine (EzWestBlue) and polyvinylidene difluoride (PVDF) membrane were purchased from ATTO Co. Ltd., Japan. The standard protein marker mixture for sodium dodecyl sulphate-polyacrylamide gel electrophoresis (SDS-PAGE), and pyridylamine (PA) labeled oligosaccharides were obtained from Takara Bio Co. Inc., Japan and Masuda Chemical Co. Ltd., Japan, respectively. Trypan blue (0.5% w/v) was purchased from NACALAI TESQUE Inc., Kyoto, Japan. RPMI 1640 medium was obtained from Nissui Pharmaceutical Co. Ltd., Japan. Fetal calf serum was purchased from Life Technologies Co. Ltd., USA. Penicillin-streptomycin was obtained from Roche Diagnostics KK., Japan.

### 2.2. Purification of Lectin

Sponges were cut into pieces by stainless steel scalpels and homogenized with 10 volumes (w/v) of tris-buffered saline (TBS) (10 mM tris(hydroxymethyl)aminomethane-HCl plus 150 mM NaCL, pH 7.4) that contained 10 mM of a protease inhibitor mixture. Homogenates were filtered through gauze and filter paper in a glass funnel and centrifuged at 14,720 × *g* in 500 mL centrifuge bottles for 1 h at 4 °C in a Suprema 21 centrifuge equipped with an NA-18HS rotor (TOMY Co. Ltd., Japan). The supernatant was centrifuged again at 27,500 × *g* in 50 mL centrifuge tubes to remove debris. The crude supernatant was applied to a chitotriosyl-agarose (5 mL) affinity column connected to Sephadex G-75 and lactosyl-agarose columns to remove HOL-30 (5 mL each), which served as the filter and trap of another lectin, respectively. The chitotriose-agarose column was washed with TBS, the lectin was eluted with 50 mM D-GlcNAc in TBS from the column and 1 mL of each fraction was collected using an automatic fraction collector. The chromatography steps during the wash and elution were monitored using an UV monitor (ATTO Co. Ltd., Japan) that measured absorbance at 280 nm and by SDS-PAGE [15]. The eluted fractions were dialysed against a volume of TBS to remove free sugar. Quantification of the lectin was determined using the BCA protein assay kit with bovine serum albumin as the standard protein. The absorbance at 562 nm [16,17] was measured using an ND-1000 spectrophotometer (Nano Drop Co. Ltd., USA).

### 2.3. Determination of Molecular Mass Using Gel Permeation Chromatography and SDS-PAGE

The purified lectin was subjected to gel permeation chromatography using a Superdex 75 column (1.0 × 32 cm) connected to an HPLC system that consisted of a PU-2089 intelligent pump and a UV-2027 UV-Vis detector (JASCO Co., Japan). The standard molecular marker proteins and the lectin were separated at a flow rate of 0.5 mL/min. Each protein was detected at an absorbance of 280 nm. To determine polypeptide size, the lectin was mixed with the sample buffer (20 mM Tris-HCl, 0.2% SDS and 10% glycerol in the presence or absence of 2% 2-mercaptoethanol; pH 6.8) and heated at 70 °C for 30 min. Aliquots of 30 μL were applied to the wells of a mini-slab gel (gel size: 80 × 100 × 1 mm; 15% and 5% polyacrylamide were used in the separation and stacking gels, respectively). The purified lectin was treated in the sample buffer at 70 °C for different times (ranging between 5 to 60 min). The molecular mass of the polypeptide was determined using SDS-PAGE at a constant current of 30 mA for 1 h [15]. After electrophoresis, the gel was stained with 0.1% (w/v) CBB R-250 in 40% methanol and 10% acetic acid, followed by de-staining with 40% methanol and 10% acetic acid. One nanomol of purified lectin was subjected to analysis of the *N*-terminus amino acid sequence using automated Edman degradations by a gas phase protein sequencer, Shimadzu model PPSQ-31A (Shimadzu Corp., Japan), according to a previously described method [11]. The cleavage of the polypeptide into peptide fragments was attempted by using trypsin, lysylendopeptidase and cyanogen bromide according to the manufacturer’s instructions.

### 2.4. Detection of Saccharides in Polypeptide of the Lectin

Sponge lectin that was separated by SDS-PAGE was electrotransferred to a PVDF membrane via the semi-dry method [18]. Lectin blotted onto PVDF membrane was soaked in TBS containing 0.05% Tween-20 at room temperature and incubated with HRP-conjugated lectins (2–5 μg/mL) for 1 h. HRP reaction was used to stain with 3,3′,5,5′-tetramethylbenzidine according to the manufacturer’s instructions [19].

### 2.5. Hemagglutination and Carbohydrate-Binding Specificity

Hemagglutination assays were performed using microtiter plates with V-bottom wells by the 2-fold serial dilution method using erythrocytes of human type A, B, O and rabbit. Carbohydrate-binding specificity of the lectin was evaluated as follows: Twenty microliters of 1% (w/v) trypsin-treated and 0.25% glutaraldehyde-fixed human type A erythrocytes (1% in TBS) were mixed with the serially diluted lectin. After incubation for 1 h at room temperature, the plates were read. One unit of hemagglutination activity was defined as the highest dilution of lectin that yielded a complete hemagglutination (HA unit). For the inhibition tests, a dilution of purified lectin to 16 units of HA was used. The inhibition of hemagglutination was assayed using 2-fold serial dilutions of the following saccharides: D-Gal, Me-α- and Me-β-D-GalNAc, Me-α- and Me-β-D-GlcNAc, D-Glc, D-Man and L-Fuc. BSM, fetuin and PSM were used at concentrations of 50 mM and 1 mg/mL [20].

### 2.6. Effects of Divalent Cations, Sulfhydryl Preservation Reagent, Temperature and pH

Two hundred and fifty-six (2^8^) hemagglutination units (HU) (about 0.5 μg/mL) of diluted lectin were tested for hemagglutination activity by the 2-fold serial dilution method in the presence or absence of divalent cations (CaCl_2_, MnCl_2_ and MgCl_2_), chelating agents (EDTA and EGTA) and sulfhydryl preservative (2-mercaptoethanol) reagents (10 mM of each reagent was used). To investigate the tolerance of the lectin to temperature and pH, it was tested over a temperature range from 20–80 °C at 10 °C increments and pH 3–13 for 1 h. Lectin was dissolved in 100 mM sodium acetate-acetic acid (pH 3–5), phosphate buffer (pH 6–7), Tris-HCl (pH 8–8.5) or glycine-NaOH (pH 9–13) at 25 °C and subsequently dialyzed against TBS (pH 7.4). Hemagglutination activity was measured in the titer plates as described above.

### 2.7. Kinetic Analysis Using Surface Plasmon Resonance

PSM (100 μg/mL) dissolved in 10 mM sodium acetate (pH 4.5) was coupled on a carboxymethyl dextran matrix of a sensor chip CM5 using a ligand coupling kit according to the manufacturer’s instructions. Remaining uncoupled activated residues on the chip were masked with 1 M neutralized ethanolamine (pH 8). Different concentrations of purified lectin (0–100 μM) in the HBS buffer were applied to the surface of a PSM-conjugated sensor chip using an auto-sampler and the association and dissociation of the lectin to immobilized PSM on the chip were analyzed for 2 min at a flow rate of 20 μL/min in 25 °C [21]. After application to the sensor chip for the association analysis, the bound lectin was washed with HBS buffer and finally it was removed from the surface of the sensor chip using 200 mM D-GlcNAc containing HBS. The transition of association to dissociation of the lectin to the PSM on the sensor chip was monitored optically as a sensorgram. The dissociation constant estimated by the association rate (*k_ass_*) and dissociation rate (*k_diss_*) of each concentrated lectin was determined using BIAevaluation software version 3.0.

### 2.8. Glycan-Binding Property by FACT

The purified lectin (1.7 mg) was dissolved in 0.1 M NaHCO_3_ (pH 8.3) containing 0.5 M NaCl, then coupled to a NHS-activated Sepharose 4 Fast Flow matrix (250 μL) overnight at 4 °C. After coupling, the uncoupled groups on the gel were masked overnight by 1 M ethanolamine-HCl (pH 8). The lectin-immobilized Sepharose gel was washed with TBS and packed into a miniature column (200 μL). The column was then connected to an HPLC system consisting of a pump (PU-2089, Jasco Co. Ltd., Japan), a fluorescence detector (FP-2020 Plus, Jasco Co. Ltd., Japan) and a data processing integrator to control the systems (ChromNAV, Jasco Co., Ltd., Japan). Sixteen PA-saccharides containing two monosaccharides of PA-GlcNAc and PA-GalNAc were manually applied to the HOL-18 conjugated affinity column at a flow rate of 250 μL/min at 20 °C [14]. PA-rhamnose was used as the inert control. All PA-saccharides were applied into the column at a concentration of 0.6 pmol in 1.6 mL. The front elution volume of the PA-saccharides was detected by measuring the fluorescence detector at 310 nm (excitation) and 380 nm (emission). The retardation volume (*V* − *V*_0_) of each PA-saccharide (*V*) compared to the inert controls of PA-rhamnose (*V*_0_) was estimated from the conversion of a bar graph. FACT was performed with TBS containing 1 mM EDTA. Analysis was conducted in triplicate in each PA-oligosaccharide and average values were indicated as bars.

### 2.9. The Cytotoxicity

Jurkat leukemia T cells and K562 erythroleukemia cells were cultured in RPMI 1640 medium (Nissui Pharmaceutical Co. Ltd., Tokyo, Japan) supplemented with 10% fetal bovine serum (FBS), penicillin (100 U/mL), and streptomycin (100 μg/mL) and maintained at 37 °C in a 95% air and 5% CO_2_ atmosphere. The cytotoxicity of the lectin was determined using the trypan blue exclusion assay with 0.5% trypan blue solution, according to a previously reported protocol [22]. The cells (2 × 10^4^; 90 μL) were seeded onto 96-well, flat-bottomed plates and treated with 1–25 μg/mL of the lectin at 37 °C for 24 h. The dead cells were counted following staining with trypan blue using a manual cell counter. The termination of cell death by the lectin (25 μg/mL) was tested in the co-presence of D-GlcNAc, D-GalNAc and D-Man (50 mM each). Results are reported as the mean of percentage standard error. Two-tailed Student’s *t*-tests were used as appropriate for statistical analysis. Only *P* values < 0.05 were considered to be statistically significant.

## 3. Results and Discussion

### 3.1. Purification of HOL-18

**Figure 1:**
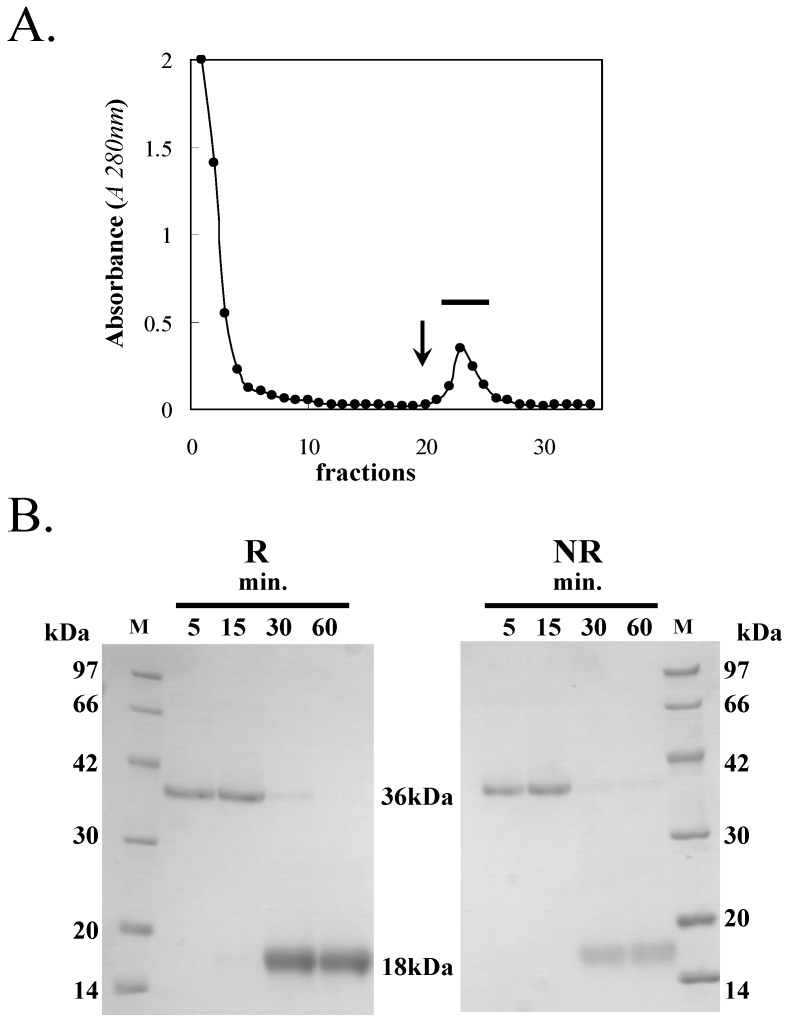
Purification of HOL-18. (**A**) The crude extract of *H. okadai* (200 g) was applied to the chitotriose-agarose column that had been equilibrated with tris-buffered saline (TBS). The column was washed with TBS and eluted with a TBS solution containing 50 mM D-GlcNAc (arrow). Fractions were collected and dialysed (bar); (**B**) HOL-18 (10 μg) was subjected to SDS-PAGE under reducing (R) and non-reducing (NR) conditions. The numbers under the bar indicate how long (5–60 min) the solution was treated at 70 °C. The following standard marker proteins (M) were used: Phosphorylase b (97 kDa), bovine serum albumin (66 kDa), ovalbumin (42 kDa), carbonic anhydrase (30 kDa), trypsin inhibitor (20 kDa) and lysozyme (14 kDa).

**Figure 2:**
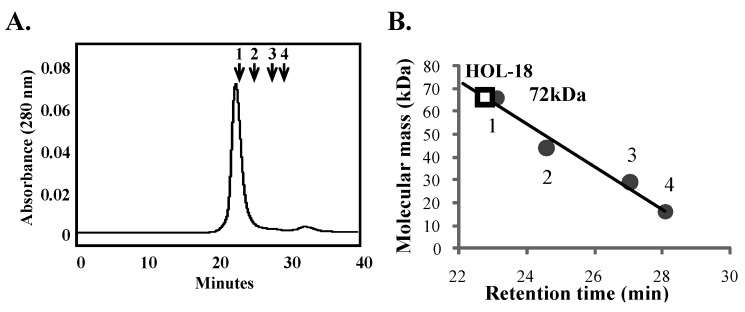
GPC and the calibration curve used to determine the molecular mass of HOL-18. The purified lectin (20 μg) was separated by HPLC on a Superdex 75 column at a flow rate of 0.5 mL/min. (**A**) The numbers indicate the standard molecular mass marker proteins used: (1) bovine serum albumin (66 kDa); (2) ovalbumin (42 kDa); (3) carbonic anhydrase (30 kDa) and (4) myoglobin (17 kDa); (**B**) The calibration curve to determine the molecular mass of the lectin. Square on the curve indicates the evaluated molecular mass of HOL-18 under native conditions.

The supernatant prepared by homogenization of *H. okadai* with TBS had a strong hemagglutination activity against trypsinized glutaraldehyde fixed-human (type-A) and rabbit erythrocytes. The crude extract was applied to the chitotriose-agarose column and the lectin was specifically eluted with D-GlcNAc-containing TBS (Figure 1A). Using SDS-PAGE under non-reducing and reducing conditions (70 °C for 30 min), the relative molecular mass of the lectin was determined to be 18 kDa (Figure 1B). The 18 kDa chitotriose-binding lectin from *H. okadai* was consequently defined as HOL-18. Interestingly, this lectin appeared as a molecular mass of 36 kDa, indicating a dimeric form. This dimeric form was retained under heat treatment for only 15 min under either reducing or non-reducing conditions; however, the dimer dissociated within 20–30 min of treatment. It suggested that these two polypeptides of HOL-18 tightly and non-covalently associate to each other. In total, 6 mg of the lectin was successfully purified from 800 g (wet mass) of sponge (Table 1). GPC of HOL-18 indicated a mass of 72 kDa, indicating that the lectin is a tetramer that is non-covalently bonded to four 18 kDa polypeptides (Figure 2). No PTH-amino acid signal was detected by direct sequencing, which suggested that the *N*-terminus amino acid of the polypeptide was blocked (data not shown). This is different from the case of HOL-30, which was suitably cleaved and sequenced into peptides by endoprotease, the generation of peptides from HOL-18 was not successful by treatment with proteases and cyanogen bromide.

**Table 1:** Purification of HOL-18.

Steps	Titer (HU)	Volume (mL)	Total activity ^a^	Protein conc. (mg mL^−1^)	Specific activity ^b^	Purification ratio (fold)	Recovery of activity (%)
Crude extract obtained by TBS	1024	200	204,800	2.3	2.2	1	100
Chitooligo-agarose	4096	10	40,960	0.7	585	292	20

^a^ Total activity is shown by Titer × volume; ^b^ Specific activity is shown by titer/mg of protein.

**Figure 3:**
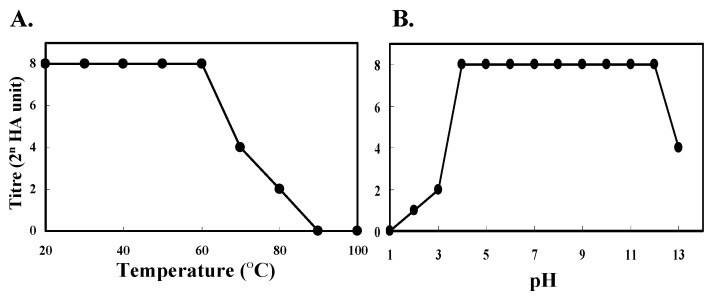
Thermostability and pH stability of the lectin. (**A**) Effects of temperature; (**B**) Effects of pH on hemagglutination activity (vertical axis indicates the titer [2^n^ HA units]).

The hemagglutination activity of HOL-18 was shown to be 1024 and 512 HU to human type-A and rabbit erythrocytes, respectively. However the lectin showed 64 HU to human type-B and type-O erythrocytes (data not shown). HOL-18 maintained maximum activity upon treatment at 60 °C (Figure 3A) and pH 4–12 (Figure 3B). The activity of HOL-18 was not enhanced even in the presence of 10 mM divalent metals or inhibited by the addition of chelating reagents such as EDTA and EGTA (data not shown). The addition of a sulfhydryl preservation reagent (2-mercaptoethanol) did not interfere with the hemagglutination activity of the lectin (data not shown). These results indicate that the activity was independent of the presence of Ca^2+^, Mn^2+^, Mg^2+^ or sulfhydryl preservation reagents.

### 3.2. Saccharide-Binding Specificity of HOL-18

The hemagglutination activities of the lectin were effectively inhibited by chitotriose (0.4 mM). These activities were also inhibited by mono-, disaccharide and glycoproteins, including methyl α-D-GlcNAc and methyl β-D-GlcNAc (3.13 mM), methyl α-D-GalNAc (12.5 mM), methyl α-D-GalNAc (6.25 mM), methyl β-D-GalNAc (12.5 mM), chitobiose (0.8 mM), PSM (0.01 mg/mL), BSM (0.125 mg/mL) and fetuin (0.125 mg/mL). The lectin recognized well GlcNAc and GalNAc but it did not selectively recognize their α- and β-anomers. On the other hand, D-Gal, D-Glc, L-Fuc, D-Man, sucrose and lactose did not result in inhibition even at concentrations over 100 mM (Table 2).

**Table 2:** Saccharide and glycoprotein specificity of HOL-18.

Saccharides	Minimum inhibitory concentration (mM)
Chitobiose	0.8
Chitotriose	0.4
D-Galactose	N.I. ^a^
Methyl α-*N-*Acetyl D-glucosamine	3.13
Methyl β-*N-*Acetyl D-glucosamine	3.13
Methyl α-*N*-Acetyl D-galactosamine	6.25
Methyl β-*N*-Acetyl D-galactosamine	12.5
D-Glucose	N.I.
D-Mannose	N.I.
L-Fucose	N.I.
Lactose	N.I.
Sucrose	N.I.
Glycoproteins	Minimum inhibitory concentration (mg/mL)
PSM	0.01
BSM	0.125
Fetuin	0.125

^a^ Inhibition did not occur even at 100 mM. Titer unit of lectin was previously adjusted to 16.

### 3.3. Glycan Moiety of HOL-18 Polypeptide

**Figure 4:**
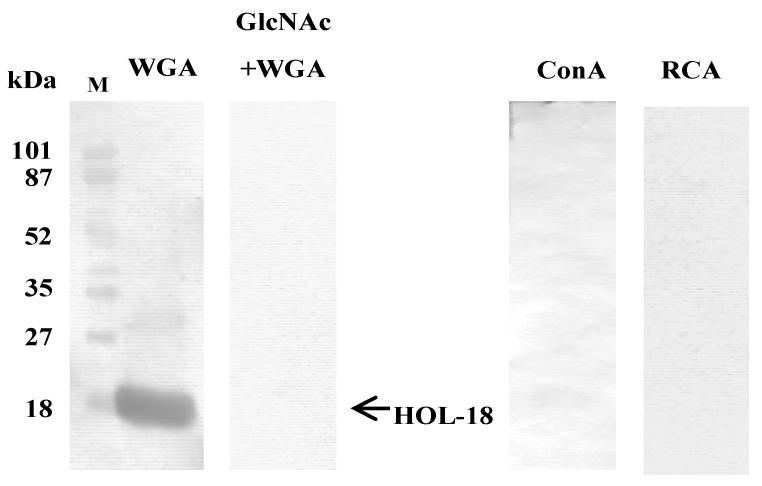
HRP-conjugated lectin blot analysis. HOL-18 (5 μg each) was subjected to SDS-PAGE and blotted onto PVDF membrane. Oligosaccharide of HOL-18 polypeptide was detected by HRP-conjugated WGA, ConA and RCA120 (1 μg each). Column GlcNAc + WGA was co-incubated with GlcNAc (50 mM) in addition to WGA for the cancellation test. M stands for molecular marker. Arrow indicates the position of HOL-18.

HRP-WGA selectively stained PVDF membrane blotted on HOL-18 and the stain was completely blocked by including 50 mM D-GlcNAc-containing TBS (Figure 4). However, other HRP-conjugated lectins (ConA and RCA120) did not stain. It indicated that the polypeptide of HOL-18 contains a covalently extended saccharide chain(s) containing D-GlcNAc residue.

### 3.4. Association and Dissociation Rates of the Lectin

**Figure 5:**
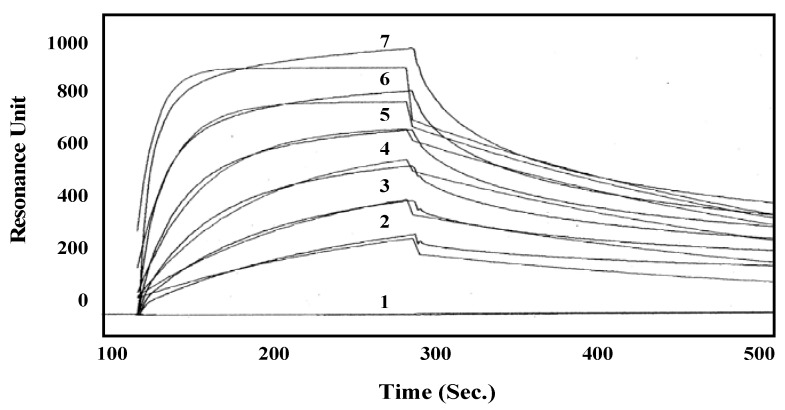
Kinetic analysis of HOL-18 by SPR. HOL-18 was applied to the CM5 sensor chip and coupled with PSM at a concentration of 5.8 ng/mm^2^. HOL-18 (0, 2, 4, 6, 8, 10 or 12 nM of each are represented by numbers 1–7 respectively) was applied onto the PSM-conjugated sensor chip at a concentration of 20 μL/min for 2.5 min. The chip was then washed with HBS for 2.5 min. Horizontal axis: Time (s); vertical axis: Resonance unit (indicating the association and dissociation rates of the analyte). The kinetics was analyzed using BIEvaluation software, version 3.0.

PSM was immobilized on the CM5 sensor chip at 5800 resonance units (RU), and using BIAcore 3000, several concentrations of HOL-18 were applied as an analyte to determine the dissociation constant of HOL-18. SPR analysis indicated that free HOL-18, when used as an analyte, binds to the immobilized PSM on the sensor chip in a dose-dependent manner (Figure 5). The kinetic parameters of the association (*k_ass_*) and dissociation rates (*k_diss_*) were calculated from the sensorgram response and simulated to mirror a 1:1 affinity model using BIAevaluation software version 3.0. The *k_ass_*, *k_diss_* and *K_D_* values of HOL-18 were calculated as 1.4 × 10^3^ M^−1^s^−1^, 6.1 × 10^−3^s^−1^ and 4.4 × 10^−6^ M, respectively. These results indicate that HOL-18 both quickly associates and dissociates from PSM, which is similar to other general lectins.

### 3.5. The Glycan-Binding Property

**Figure 6:**
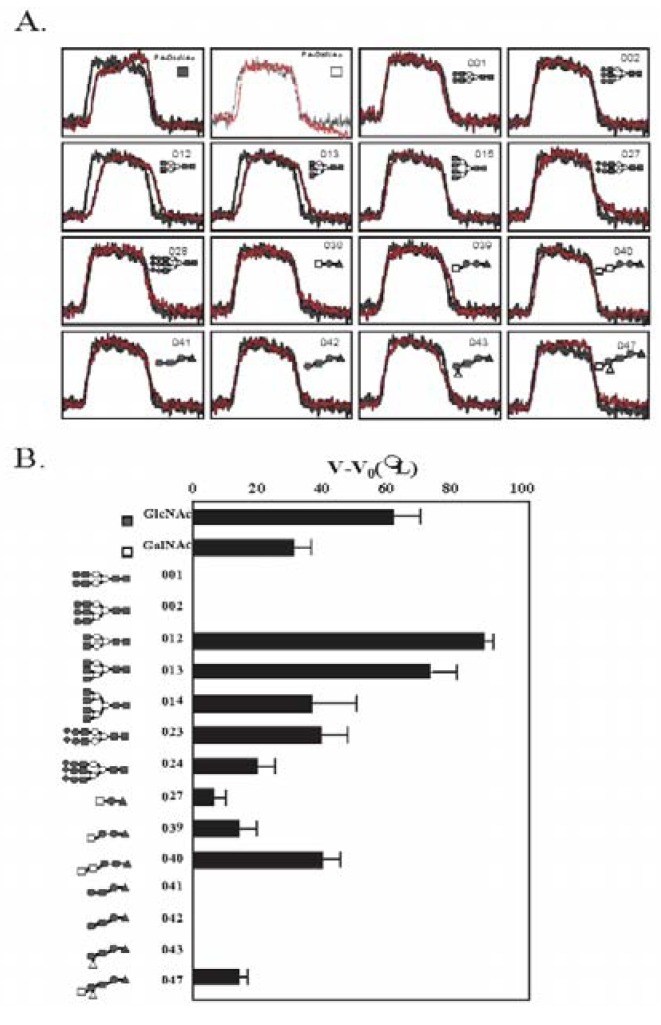
Glycan-binding profile of HOL-18. (**A**) Elution curves of each PA-oligosaccharide (listed in Table 3) obtained from the HOL-18-immobilized column; (**B**) Bar graph representation of the *V* – *V*_0_ of HOL-18 toward PA-glycans. PA-rhamnose was used as the inert control. Schematic representations of the PA-oligosaccharide for the analysis. Symbols for ▲, ○, ●, ■, □, △ and ◆ represent pyranose rings of D-Glc, D-Man, D-Gal, D-GlcNAc, D-GalNAc, L-Fuc and D-NeuAc, respectively (anomeric carbon, *i.e*., position 1, is placed at the right side, and 2, 3, 4, 5 and 6 are placed clockwise from positon C-1). Thick and thin bars represent α- and β-linkages, respectively (See Table 3). Results were repeated triplicates and reported as the mean of standard error.

To determine the glycan-binding profile of HOL-18, 14 different PA-oligosaccharides, PA-GlcNAc and PA-GalNAc were used (Table 3). HOL-18 has shown strong affinities to branched N-acetylated glycans of complex-type oligosaccharides in the following hierarchy: Bi-antennary (**012**) > tri-antennary (**013**) > tetra-antennary (**014**). Sialylated complex-type oligosaccharides (two-branched **023** and three-branched (**024**) with *N*-acetyl group also interacted with HOL-18. With regards to glycosphingolipid-type glycans, Forssman pentasaccharide, which contains two GalNAc residues (**040**), blood type A hexasaccharide that contains an α-GalNAc residue (**047**) and asialo-GM2-tetrasaccharide with β-GalNAc (**027**) were also weakly recognized by the lectin (Figure 6A). Based on the results of FACT, the glycan-binding properties of the lectin indicated that HOL-18 is able to recognize serum glycoprotein-type oligosaccharides that contain D-GlcNAc (agalactosyl) and D-Neu5Ac (sialosyl) clusters and glycosphingolipid oligosaccharides having D-GalNAc at the non-reducing termini (Figure 6B) in addition to *O*-linked oligosaccharide derived from mucin-type glycoprotein (Table 2).

**Table 3:** List of PA-oligosaccharides for FACT analysis of HOL-18.

PA No.	Glycoprotein type PA-oligosaccharide	PA No.	Glycosphingolipid type PA-oligosaccharide
[001]	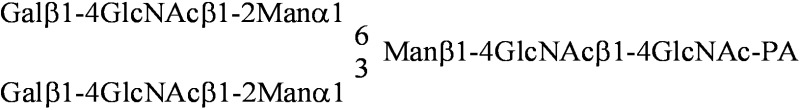	[027]	GalNAcβ1-4Galβ1-4Glc-PA
[002]	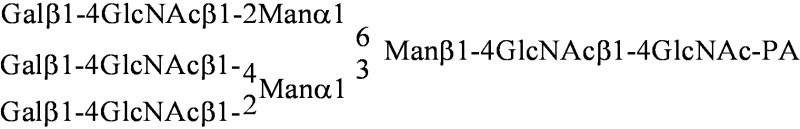	[039]	GalNAcβ1-3Galα1-4Galβ1-4Glc-PA
[012]	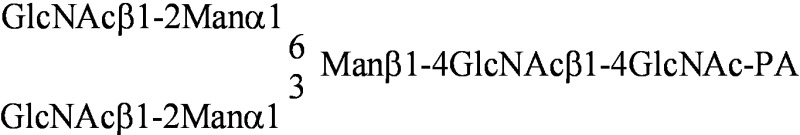	[040]	GalNAcα1-3GalNAcβ1-3Galα1-4Galβ1-4Glc-PA
[013]	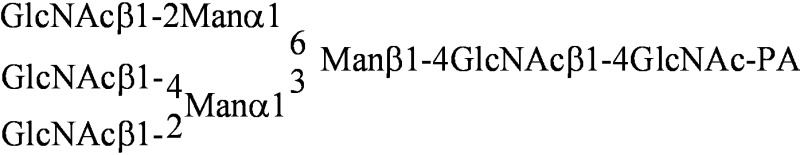	[041]	Galβ1-4GlcNAcβ1-3Galβ1-4Glc-PA
[014]	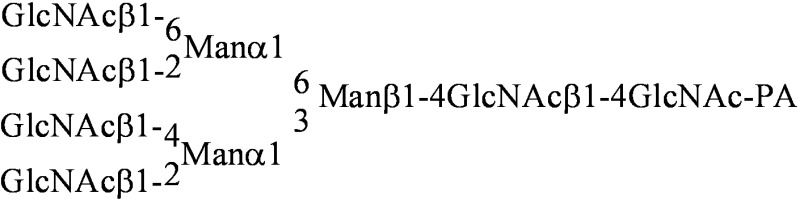	[042]	Galβ1-3GlcNAcβ1-3Galβ1-4Glc-PA
[023]	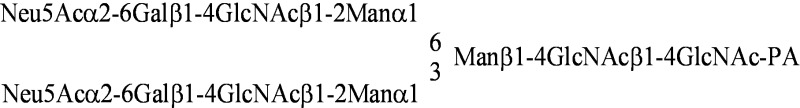	[043]	Fucα1-2Galβ1-3GlcNAcβ1-3Galβ1-4Glc-PA
[024]	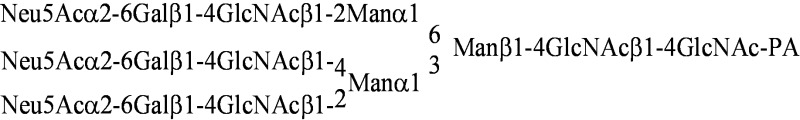	[047]	GalNAcα1-3(Fucα1-2)Galβ1-3GlcNAcβ1-3Galβ1-4Glc-PA

### 3.6. Glycan-Dependent Cytotoxicity

**Figure 7:**
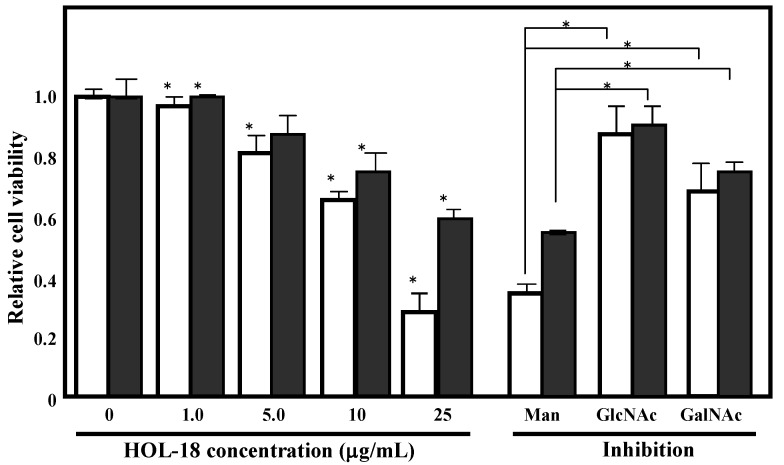
Glycan-dependent cytotoxicity of HOL-18 against Jurkat and K562 cells. Cell viability was determined by using the Trypan blue exclusion assay. Cells were treated with HOL-18 (0–25 μg/mL). White and gray bars indicate cell viability of the Jurkat and K562 cells, respectively. Specific inhibition of the cell viability reduction activity of HOL-18 by the addition of saccharides (Man: Mannose, GlcNAc: *N*-acetyl-D-glucosamine, GalNAc: *N*-acetyl-D-galactosamine. 50 mM of saccharides were added to each experiments.). Error bars represents the mean ± SE, *n* = 3. Only *P* values < 0.05 were considered to be statistically significant indicated by asterisk (*).

HOL-18 (at concentrations of 1–25 μg/mL) showed 40% and 50% cell death against T-cell leukemia Jurkat and erythroleukemia K562 cells, respectively, as determined by the trypan blue exclusion assay (Figure 7). The cytotoxic activity against both Jurkat and K562 cells of HOL-18 were considerably inhibited by the co-presence of 50 mM D-GlcNAc and D-GalNAc in the medium (Figure 7; column GlcNAc and GalNAc). 

HOL-18 had curious biochemical properties in that the 18 kDa polypeptide formed a non-covalent dimer (32 kDa) whose integrity was tightly maintained even if the lectin was heated to 70 °C for 15 min under both reducing and non-reducing conditions (Figure 1B). Finally, it exhibits a tetrameric form (72 kDa) in native conditions (Figure 3). Lectin-glycome analysis showed that HOL-18 was demonstrated to bind to two-branched agalactosyl or sialyl complex-type oligosaccharides of glycoprotein, globo- and lacto-series oligosaccharides such as Forssman and blood type A antigens containing α-D-GalNAc of glycosphingolipid, respectively. 

According to the binding profile, HOL-18 showed cytotoxicity to both T-cell leukemia (Jurkat) and erythroleukemia (K562) cells which are known to express D-GlcNAc and D-GalNAc residues, in addition to D-NeuAc, at the non-reducing termini of oligosaccharides derived from cell membrane glycoproteins [23]. From the view of the hemagglutination inhibition test, it appeared that the lectin was inhibited by such glycoproteins as PSM, BSM and fetuin (Table 2). These results indicated that HOL-18 has an affinity for O-linked oligosaccharides in mucins and serum glycoproteins. PSM possesses non-sialylated oligosaccharides, such as GlcNAcβ1-6 (±Fucα1-2Galβ1-3)GalNAc and GlcNAcβ1-6GalNAc. Conversely, BSM possesses Neu5Acα2-6(±GlcNAcβ1-3)GalNAc in addition to sialyl Tn-antigen (Neu5Acα2-6GalNAc) [24]. Fetuin contains O-linked oligosaccharides and also contains sialyl *N*-acetyllactosamine (Neu5Acα2-3/6Galβ1-4GlcNAc) chains in complex-type *N*-linked glycans [25].

Recently, a sponge lectin with chemotactic activity toward murine neutrophils was reported and it exhibited *N*-acetyl sugar-binding activity similarly to HOL-18 [8]. This suggested that the lectin might have cell regulatory functions in addition to toxicity. Chitin and Glycosphingolipids having *N*-acetyl sugars have been recently isolated from different marine sponges [26,27], which may be binding partner candidates for HOL-18. 

## 4. Conclusions

A lectin, HOL-18 that consisted of tetrameric 18 kDa subunits was purified from the Japanese demosponge *H. okadai* and its glycan-binding affinity toward oligosaccharides with *N*-acetylated sugars was elucidated by a lectin-glycome study. HOL-18 was cytotoxic to cancer cells through a lectin-glycan interaction. 
